# Sorting the mind: A systematic review and meta-analysis protocol of transcutaneous auricular vagus nerve stimulation on cognitive functions

**DOI:** 10.1371/journal.pone.0347849

**Published:** 2026-04-24

**Authors:** Fangqing Liu

**Affiliations:** Faculty of Biology, Medicine and Health, The University of Manchester, Manchester, England, United Kingdom; Ritsumeikan Asia Pacific University: Ritsumeikan Asia Taiheiyo Daigaku, JAPAN

## Abstract

**Background:**

Transcutaneous auricular vagus nerve stimulation (taVNS) is a non-invasive neuromodulation technique that activates vagal afferents projecting to prefrontal–limbic circuits implicated in attention, memory, and emotion regulation. Preliminary studies suggest that taVNS may enhance cognitive performance; however, the evidence remains fragmented across domains and populations.

**Objectives:**

This systematic review and meta-analysis aim to (1) quantify the overall effects of taVNS on cognitive functions, (2) examine its efficacy across clinical and non-clinical populations, and (3) identify moderators influencing variability in outcomes, including stimulation parameters, participant characteristics, and study design features.

**Methods:**

Following PRISMA-P guidelines, this protocol will be prospectively registered with PROSPERO. Six databases (PubMed, EMBASE, PsycINFO, Web of Science, CENTRAL, and Scopus) and major trial registries will be systematically searched. Eligible studies include randomised controlled trials assessing validated cognitive outcomes following taVNS compared with sham or active controls. Effect sizes will be calculated as Hedges’ g and pooled using random-effects models. Heterogeneity will be evaluated with I² and τ statistics; moderator and meta-regression analyses will explore dose–response and population effects. Risk of bias will be assessed with RoB 2 for randomised trials and ROBINS-I for non-randomised studies, and the certainty of evidence will be rated using GRADE separately by study design.

**Expected Results and Conclusions:**

This review will provide the first quantitative meta-analytic synthesis of taVNS-induced cognitive modulation across executive, attentional, affective, and learning domains in both clinical and healthy populations, complementing recent narrative syntheses by offering pooled effect size estimates, formal heterogeneity assessment, and a GRADE-rated evidence hierarchy. By delineating domain-specific efficacy and optimal stimulation parameters, the findings aim to inform future clinical applications and the development of standardized neuromodulation protocols.

## Introduction

Our exploration into enhancing cognitive functions has never ceased. Previous cognitive improvement methods primarily relied on traditional mental training and educational interventions ([1];[2];[3]), aiming to elevate an individual’s cognitive abilities through repetitive practice and memory reinforcement. However, as our understanding of brain structure and function deepens, we have discovered that cognitive functions are not solely dependent on knowledge accumulation and practice but is also profoundly influenced by neurobiological factors. This has led us to gradually expand our research perspective from external environmental factors to the neural mechanisms within the brain when exploring pathways for cognitive enhancement.

The past two decades have witnessed an increasing emphasis on neuromodulation as a strategy to address core deficits in disorders that remain refractory to conventional care (see [4]). Unlike pharmacotherapy or psychotherapy, neuromodulation acts on neural circuits implicated in dysfunctional states, though for peripheral approaches such as taVNS the effects are substantially more diffuse than this phrasing implies via controlled electrical or magnetic stimulation, offering the prospect of more rapid, mechanistically grounded, and durable change in brain–cognition relationships ([5];[6]). Among non-invasive options, Transcranial magnetic stimulation (TMS) and transcranial direct current stimulation (tDCS) enable direct modulation of neural activity in specific brain regions, thereby enhancing cognitive functions ([7];[8]). These non-invasive neuromodulation techniques, which alter neuronal activity patterns through electrical currents or magnetic fields, have demonstrated potential in multiple studies to enhance working memory, attention, language abilities, and emotional regulation ([9];[10]). They have shown particularly positive effects in treating cognitive impairments in Alzheimer’s disease [7], depression [11], and attention deficit hyperactivity disorder (ADHD) [12]. Both techniques, however, have limited capacity to engage deeper limbic structures ([13]). At the invasive end of the spectrum, deep brain stimulation (DBS) is highly effective for treatment-resistant movement disorders and is under active study for psychiatric disorders [14], but it is resource-intensive and ethically challenging. Implanted vagus nerve stimulation (VNS) has demonstrated robust long-term benefits in epilepsy [15] and depression [16], highlighting the therapeutic potential of vagal pathways, yet the requirement of surgical implantation and risk of complications restricts its wider use.

**Fig 1:**
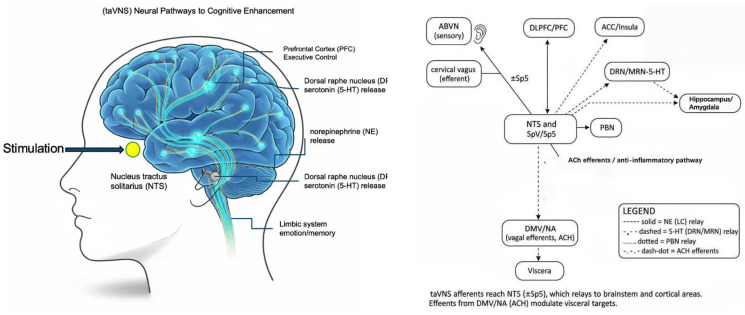
taVNS Afferent Pathways and Modulatory/Efferent Effects.

**Fig 2:**
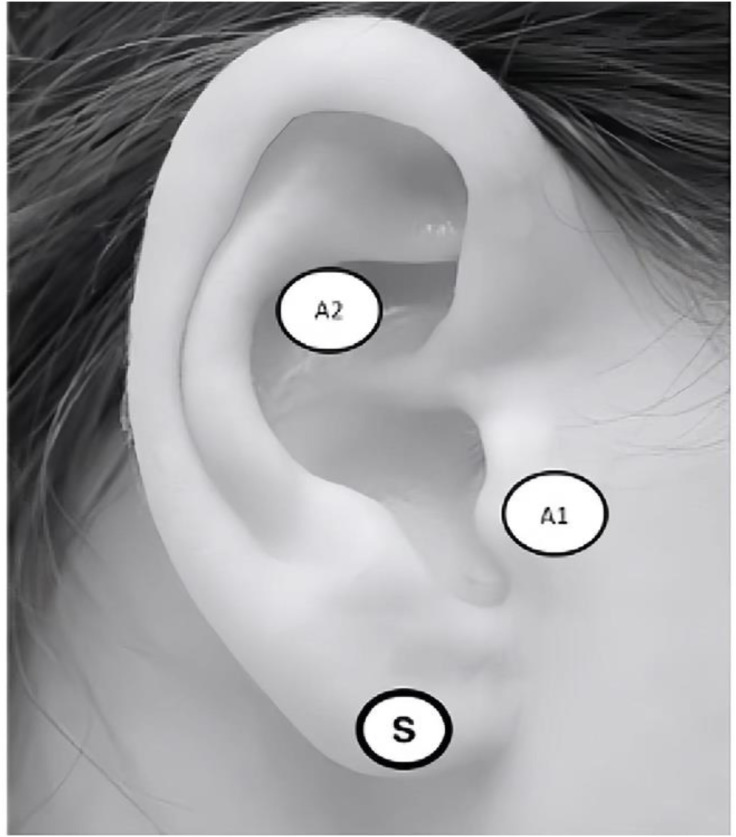
taVNS Ear Targets.

Against this backdrop, neuromodulation has reframed a familiar question: what if we could modulate the neural circuits that regulate arousal, affect, and inhibitory control—directly and non-invasively? Transcutaneous auricular vagus nerve stimulation (taVNS) has emerged as an especially compelling alternative to both invasive neuromodulation (surgical VNS, DBS) and cortically-targeted non-invasive approaches (TMS, tDCS) that cannot readily engage deeper limbic and brainstem circuitry. By stimulating the auricular branch of the vagus nerve at the external ear, taVNS provides non-invasive access to vagal afferents while avoiding surgical risks [17]. Neuroanatomical and functional studies show that these afferents terminate in the nucleus tractus solitarius (NTS), which relays to the locus coeruleus and to limbic and prefrontal regions (amygdala, hippocampus, medial prefrontal cortex) implicated in emotion regulation, autonomic balance, and executive control [18]. These projections enable taVNS to influence broad neuromodulatory systems, including noradrenergic, cholinergic, and serotonergic pathways, thereby exerting effects on arousal, attention, and affect regulation [19] (Fig 1, 2).

### Sorting the Mind: Cognitive Functions in taVNS

**Fig 3:**
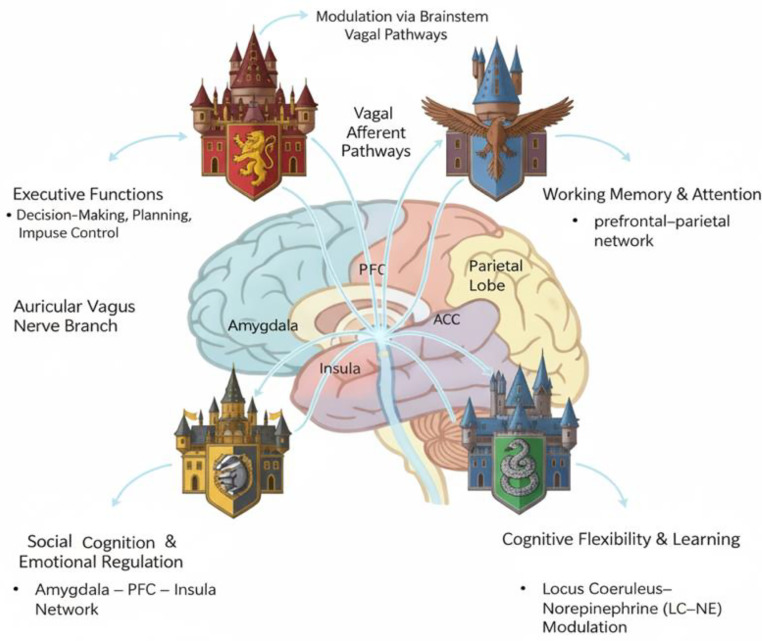
Cognitive Functions and taVNS: A Four-Domain Framework.

An increasing number of studies are now anchoring their focus on specific aspects of cognitive functions, aiming to understand the role of taVNS within these domains [20]. However, cognitive functions are not an isolated puzzle piece; it is a vast and intricate Hogwarts LEGO castle, woven from countless brain regions, neural circuits, and cognitive processes. The slightest alteration anywhere within this network can trigger systemic effects [21]. To comprehend how taVNS operates within this framework, we cannot confine ourselves to a single cognitive dimension. Much like dividing Hogwarts into four houses during our LEGO construction, this study segments cognitive functions into four major modules (Fig 3).

This four-domain taxonomy is theoretically grounded in converging evidence from cognitive neuroscience and is designed to match the specific neural targets of taVNS. (1) Executive Functions are anchored in the prefrontal cortex and its top-down control networks [22]; (2) Working Memory and Attention are jointly governed by prefrontal–parietal circuitry and the locus coeruleus–norepinephrine system [21], both of which are direct downstream targets of vagal afferents; (3) Social Cognition and Emotional Regulation share overlapping circuitry involving the amygdala, insula, and anterior cingulate cortex [23], which are regions that are selectively engaged by parasympathetic modulation; and (4) Cognitive Flexibility and Learning recruit frontostriatal loops and hippocampal plasticity mechanisms ([24];[25]), which have been shown to be sensitive to noradrenergic and cholinergic tone. The potential conceptual overlap between domains is addressed at the operational level: each cognitive measure will be assigned to a domain according to a pre-specified classification guide (available in Appendix II) that operationally distinguishes the domains by the primary cognitive process being assessed, rather than the neural substrate alone. Specifically, tasks requiring the active online updating, maintenance, or manipulation of information (e.g., N-back, Digit Span) are classified under Working Memory and Attention; tasks requiring the shifting of mental set in response to feedback or rule changes (e.g., Wisconsin Card Sorting Test, Intra-/Extra-Dimensional Shift) are classified under Cognitive Flexibility and Learning; tasks requiring the generation, monitoring, or inhibition of goal-directed actions under conflict (e.g., Stroop, Stop-Signal) are classified under Executive Functions; and tasks assessing the recognition, interpretation, or regulation of emotional or social information (e.g., Facial Emotion Recognition, Emotion Regulation Questionnaire) are classified under Social Cognition and Emotional Regulation. Any measure that plausibly spans two domains (e.g., the Trail Making Test, which involves both set-shifting and executive control) will be assigned by consensus between two raters using the primary cognitive demand criterion, and the assignment will be recorded for sensitivity analyses. This approach minimises classification ambiguity and ensures that boundary cases are handled consistently and transparently.

### Executive functions

This module governs advanced cognitive activities such as decision-making, planning, problem-solving, and impulse control. Executive functions are not only important for individuals navigating complex environments but also represent the core capacity for flexible adaptation and adjustment to change [22]. They enable individuals to demonstrate qualities of “resolve” and ‘initiative’ when confronting intricate decisions. Within taVNS research, how these core cognitive functions are influenced—particularly self-regulation under high-pressure scenarios— emerges as a critical question. The present synthesis will quantify whether executive function outcomes improve following taVNS; however, we acknowledge that even a positive pooled effect would not resolve questions about the precise neural mechanisms (e.g., whether prefrontal modulation of impulse control is specifically responsible), and conclusions about mechanism will accordingly be drawn with appropriate caution.

### Working memory and attention

Working memory represents the mental workspace that allows individuals to temporarily store, update, and manipulate information essential for reasoning, comprehension, and goal-directed behaviour [26]. It is not merely a passive holding system but a dynamic process that integrates incoming sensory input with existing knowledge to facilitate complex cognitive operations. Attention, on the other hand, functions as the selective gatekeeper of cognition—allocating limited neural resources toward task-relevant stimuli while inhibiting distractions [27]. From a neurocognitive perspective, these two mechanisms are deeply interwoven. The central executive component of working memory relies heavily on attentional control systems mediated by the prefrontal cortex and parietal networks ([21];[28]). This interaction enables individuals to maintain focus under conditions of high cognitive load and to flexibly shift attention when task demands change [29].

### Social cognition and emotional regulation

Social cognition encompasses a constellation of mental processes that enable individuals to perceive, interpret, and predict others’ emotions, intentions, and social behaviours [30]. It forms the cognitive foundation for empathy, moral reasoning, and adaptive interpersonal functioning [30]. Emotional regulation, by contrast, refers to the capacity to monitor, evaluate, and modify one’s own emotional reactions to maintain psychological homeostasis and achieve social harmony [31]. Together, these systems constitute the core of affective intelligence—an essential element of prosocial behaviour and interpersonal sensitivity.

From a neurobiological perspective, both social cognition and emotional regulation are supported by overlapping neural circuits involving the prefrontal cortex, amygdala, insula, and anterior cingulate cortex [23]. These regions mediate the intricate balance between emotional reactivity and cognitive control [32]. taVNS, by modulating vagal afferent pathways and enhancing parasympathetic tone, may exert a dual influence: enhancing emotion recognition accuracy and improving self-regulatory capacity. The presumed direction of enhancement (rather than impairment) is grounded in evidence that increased parasympathetic tone, which taVNS reliably produces, is associated with improved vagally-mediated cardiac regulation, reduced amygdala reactivity, and better prefrontal inhibitory control over limbic responses [31]. These physiological shifts are mechanistically linked to more accurate affective processing rather than disruption of it. We nonetheless treat this as a directional hypothesis to be tested; the meta-analysis will examine effects in both directions and will report the full distribution of outcomes rather than assuming uniform enhancement. Such modulation could facilitate more adaptive social responses, attenuate maladaptive emotional arousal, and enhance empathy-driven interactions.

### Cognitive flexibility and learning

Cognitive flexibility constitutes the executive faculty that enables individuals to modify their cognitive sets, shift attention, and adapt behavioural strategies when confronted with new or conflicting task demands [24]. It reflects an individual’s ability to disengage from habitual responses and adopt novel, contextually appropriate solutions—an essential mechanism for strategic reasoning and adaptive problem-solving. Complementing this, learning ability represents the progressive consolidation of knowledge and experience through iterative feedback and neural plasticity, ensuring that past encounters inform future decisions ([25]). Together, these capacities embody the essence of intellectual adaptability —the wisdom to evolve one’s strategy in pursuit of long-term goals. taVNS, by engaging vagal afferents and influencing the locus coeruleus–norepinephrine system, may enhance the neural mechanisms underlying cognitive exploration and adaptive learning. Such stimulation promotes heightened alertness, efficient error monitoring, and greater behavioural plasticity—key components of strategic cognition.

### Current evidence landscape

The burgeoning interest in taVNS, representing a rapidly evolving field within cognitive enhancement research. However, the existing research landscape remains fragmented, with disparate findings across domains. Previous meta-analyses on taVNS only investigated its effects in other domains, such as insomnia [33], pupil dilation [34], heart rate variability (HRV) [35] or its overall safety [36]. Only one previous meta-analysis has specifically examined its efficacy on cognitive functions, and that study focused on healthy individuals. Ridgewell et al. [20] reported a small but significant weighted effect size of *g* = 0.21 for overall cognitive performance following taVNS, suggesting a modest enhancement of cognition. Critically, that meta-analysis was restricted to healthy participants, and a growing body of clinical evidence published since 2021 was not captured. The specific limitations of the Ridgewell et al. [20] analysis, and the rationale for the present synthesis, are detailed in the following section.

As detailed above, the existing evidence base for taVNS effects on cognition remains limited to one study [20] that excluded clinical populations. This synthesis, while valuable, has several important limitations that motivate the present analysis. First, it exclusively enrolled healthy participants (65 clinical-sample effect sizes were excluded), leaving open whether the g = 0.21 effect generalises to patient populations in whom taVNS is most commonly used. There is no a priori reason to assume that effects observed in healthy controls transfer to individuals with, for example, Alzheimer’s disease or ADHD, whose underlying neurobiology and baseline cognitive capacity differ substantially. Second, at least one study included in that meta-analysis has since been retracted owing to problematic data reporting; the retracted effect size was among the larger estimates in the pool (d > 0.60), and its removal from the original analysis would meaningfully reduce both the pooled estimate and its precision. Third, the five years since publication have produced a substantial new body of clinical evidence across multiple cognitive domains that was unavailable to Ridgewell et al. Based on their 19 eligible effect sizes and the subsequent publication rate in this field, we estimate that the present synthesis will incorporate approximately 30 independent effect sizes, nearly doubling the evidence base and affording substantially greater power to detect domain-specific and moderator effects. For these reasons, a new, updated, and clinically focused meta-analytic synthesis is warranted.

## Research objectives

The research objective (RO)s of this study is therefore threefold:

**RO1:** Assess the overall Effect of taVNS on cognitive functions and systematically evaluate the impact of taVNS on various cognitive domains.

**RO2:** Investigate the impact of taVNS on clinical populations: to assess how taVNS affects individuals with cognitive impairments related to various conditions.

**RO3:** Examine moderator variables and heterogeneity in effect sizes: 1) to explore the role of different moderators (e.g., stimulation parameters like frequency, intensity, duration) on the effectiveness of taVNS. 2) investigating heterogeneity across studies.

## Methodology

### Protocol and registration

This systematic review and meta-analysis will follow the Preferred Reporting Items for Systematic Reviews and Meta-Analyses Protocol (PRISMA-P) guidelines and is prospectively registered in the PROSPERO (ID: CRD 420251166774). The designation “systematic review and meta-analysis” reflects a deliberate methodological decision. The systematic review component serves functions that the quantitative synthesis alone cannot fulfil: (1) it will provide a structured narrative synthesis of studies that cannot be pooled quantitatively due to insufficient statistical reporting or unique outcome operationalisations; (2) it will document the stimulation parameter landscape, identifying gaps and inconsistencies in protocol reporting that are clinically important even when effect sizes are not extractable; (3) it will map study quality and methodological trends across the literature, informing both interpretation of the pooled estimates and recommendations for future research design. Case studies are excluded because they cannot contribute effect sizes and do not include control conditions; however, the systematic review component will note prominent single-subject findings where they inform understanding of inter-individual variability. This distinction between the qualitative and quantitative contributions of the review is now maintained throughout the manuscript.

### Search strategy

A comprehensive and systematic search strategy has been developed in consultation with a specialist librarian to ensure broad coverage of biomedical, psychological, and interdisciplinary literature. The full search strings for each database, including all Boolean operators and controlled vocabulary terms (e.g., MeSH headings for PubMed), are provided in Appendix I. Briefly, the core search terms include: (“vagus nerve stimulation” OR “transcutaneous auricular vagus” OR “taVNS” OR “auricular vagal”) AND (“cognit*” OR “executive function” OR “attention” OR “memory” OR “learning” OR “emotion regulation” OR “social cognition” OR “cognitive flexibility”). We will search six electronic databases from their inception up to the date of final search execution (no end-date restriction will be applied in the search syntax; the search will be run immediately prior to PRISMA flow completion and the actual date will be reported in the final manuscript), including PubMed, EMBASE, PsycINFO, Web of Science Core Collection, Cochrane Central Register of Controlled Trials (CENTRAL), Scopus. To minimise publication bias and identify unpublished or ongoing studies, we will also systematically search trial registries, including ClinicalTrials.gov and the World Health Organization (WHO) International Clinical Trials Registry Platform (ICTRP).

In addition to database and registry searches, we will conduct extensive hand-searching procedures. The reference lists of all eligible full-text articles, as well as those of relevant systematic reviews and meta-analyses, will be examined to identify additional studies that may not have been captured through electronic searches. Grey literature will also be considered, including doctoral dissertations, and theses indexed in ProQuest Dissertations & Theses Global and WorldCat. Reports from scientific meetings and other unpublished materials will be examined where sufficient methodological detail and outcome data were available. All searches will be conducted without restrictions on languages or publication year.

### Study selection and data extraction

Inclusion and exclusion criteria are listed in Table 1 and data extracted from articles can be found in Table 2.

**Table 1:** Inclusion and exclusion criteria for study selection.

Category	Inclusion Criteria	Exclusion Criteria
**Study Type**	• Randomised controlled trials (RCTs), quasi-experimental studies, and non-randomised interventional studies (e.g., single-arm before-after trials in which all participants receive taVNS and are compared to their own baseline). Purely observational designs with no taVNS intervention are excluded by definition and will not be included. • Pre- and post-intervention designs (both longitudinal and cross-sectional). • Studies including a control group (active or sham) or reporting pre–post intervention comparisons.	• Case reports, reviews, commentaries, and editorials. • Studies without a control or baseline comparison (e.g., single-group post-intervention only, i.e., no pre-intervention baseline). • Studies that used taVNS in combination with another active intervention (e.g., cognitive training, pharmacotherapy) without a taVNS-alone or sham condition, as these designs do not permit isolation of the taVNS effect. • Studies reporting insufficient statistical information to permit effect-size calculation or estimation (i.e., studies that report no means, standard deviations, inferential statistics, or response rates from which effect sizes can be derived), and from which data cannot be obtained from authors.
**Population**	• No age or ethnic restriction.	• Animal or non-human studies.
**Intervention**	• Studies investigating transcutaneous auricular vagus nerve stimulation (taVNS) as the main intervention, regardless of stimulation parameters (e.g., intensity, frequency, duration, intervention period).	• Studies evaluating non-taVNS interventions (e.g., pharmacological treatments, other neuromodulation methods) without sufficient taVNS-specific information.
**Outcome Measures**	• Validated measures of cognitive function (e.g., self-report scales, neuropsychological tests, or behavioural tasks). • Clinical measures of cognitive impairment when includes clinical population (e.g., *Alzheimer’s Disease Assessment Scale – Cognitive Subscale*, *Wechsler Adult Intelligence Scale*).	• Studies not using objective cognitive tests or not clearly reporting relevant cognitive outcomes.
**Language**	• No language restriction (translations conducted where possible).	—
**Publication Type**	• Peer-reviewed journal articles, dissertations, conference abstracts (if sufficient methodological details provided), and registered clinical trials.	—
**Other Exclusions**	—	• Studies lacking clear description of taVNS stimulation parameters (e.g., frequency, intensity, or duration).

**Table 2:** Study characteristics and data extraction framework.

Category	Description
**Study Characteristics**	Study ID: Author(s) and publication year. Study Design: Type of study. Sample Size: Total sample and subgroup sizes for intervention and control groups. Duration of Study: Total study length and follow-up periods (if applicable). Country and Setting: Geographic location and study context.
**Participant Characteristics**	Demographics: Age, gender distribution, and other relevant variables. Clinical Population: Diagnostic category or condition (e.g., ADHD, depression, Alzheimer’s disease).
**Intervention Characteristics**	taVNS Parameters: Frequency (Hz), intensity (mA), duration per session (minutes), total intervention period. Mode of Stimulation: Auricular or transcutaneous application. Control Group/Comparison: Type of control condition (active, sham, alternative intervention, or wait-list control).
**Outcome Measures**	Primary Cognitive Domains: executive function, attention, memory, social cognition, emotional regulation, and cognitive flexibility. Specific Tests Used: e.g., *Stroop Task*, *Wisconsin Card Sorting Test*, *Digit Span*, *Emotional Regulation Scale*. Secondary Outcomes: Additional outcomes such as mood, motivation, or quality of life. Time Points of Measurement: Pre- and post-intervention, and follow-up assessments (if applicable).
**Statistical Information**	Reported effect sizes (Cohen’s *d*, Hedges’ *g*, etc.) with corresponding 95% confidence intervals. Reported significance tests (e.g., *p* values, *t* or *F* statistics).

## Quality assessment

We will evaluate quality assessment for all randomised controlled trials/ included studies using the Cochrane Risk of Bias 2 tool (RoB 2). Assessments will be conducted at the result level, i.e., separately for each prespecified outcome and time point. Two reviewers will independently complete the RoB 2 signaling questions after a calibration exercise; disagreements will be resolved by discussion. Domain-level and overall judgements will follow RoB 2 guidance: low risk of bias, some concerns, or high risk of bias. For crossover trials, we will apply the RoB 2 crossover extension, which includes assessment of period and carryover effects and adequacy of washout. Where substantial carryover cannot be excluded, we will perform a sensitivity analysis using first-period data only. For non-randomised interventional studies (e.g., single-arm before-after designs), risk of bias will be assessed using the Risk of Bias In Non-randomised Studies of Interventions (ROBINS-I) tool [37], which evaluates seven bias domains including confounding, selection, classification, deviation from intended intervention, missing data, measurement of outcomes, and selection of the reported result. Regarding the integration of GRADE certainty ratings across design types: because RCTs begin at “high” certainty and non-randomised studies begin at “low” certainty under the GRADE framework [38], we will present GRADE assessments separately for the RCT evidence body and the non-randomised evidence body rather than merging them into a single rating. This approach preserves the interpretive integrity of GRADE and avoids artificially deflating certainty for the RCT subgroup or inflating it for the non-randomised subgroup.

## Statistical analysis

### Effect size imputation and calculation

Missing data represents an inevitable challenge in meta-analysis, and we will address it systematically through a hierarchical approach. Our first step will be to contact corresponding authors of studies with incomplete reporting, allowing a two-week response window for data provision. When author contact proves unsuccessful or infeasible, we will employ statistical imputation methods. For graphically presented data, we will use digital plot digitisation software to extract numerical values from Figs. When studies report only inferential statistics such as F-values, t-values, or exact p-values, we will employ established conversion formulae to calculate standardised mean differences [39]. In cases where means are unreported, but medians and ranges are provided, we will use validated approximation methods to estimate means and standard deviations, noting these approximations in our dataset and conducting sensitivity analyses to assess their impact.

Our primary metric will be the standardised mean difference using Hedges’ g, which is preferred over Cohen’ s d for its correction for small sample bias (when sample sizes <20).

When means and standard deviations are available for both intervention and control groups, we will calculate effect sizes directly from these values. For studies reporting pre-post change scores, we will utilise these alongside correlation coefficients when available. Critically, for each extracted effect size we will record in our coding sheet whether the standardisation denominator is based on (a) the pooled post-intervention standard deviation (between-group variability) or (b) the standard deviation of change scores (within-subject variability). Effect sizes standardised on change-score variability can be substantially inflated relative to those standardised on between-group variability, because the denominator conflates the magnitude of the treatment effect with its homogeneity across participants. We will therefore conduct sensitivity analyses comparing pooled estimates computed from each class of effect sizes separately and will report these two sets of estimates alongside the overall pooled estimate so that readers can assess the impact of standardisation choice on the conclusions. In cases where only inferential statistics are provided, we will extract or convert t-statistics, F-statistics, or p-values to derive standardised mean differences. All reported effect sizes will be converted to our common metric to ensure comparability across studies.

When studies designate a primary outcome measure, we will prioritise this selection. In the absence of such designation, we will favor well-validated, standardised tests over custom or ad-hoc measures. For studies reporting multiple measures within a single cognitive domain, we will calculate composite scores by averaging effect sizes while accounting for the correlation between measures, assuming a correlation of 0.5 when specific correlation data are unreported. This default of r = 0.5 is consistent with conventional practice in psychological and cognitive meta-analyses ([40];[41]) and represents a conservative mid-point estimate of the inter-measure correlation when empirical values are unavailable. Importantly, we acknowledge that this assumption can meaningfully influence pooled composite estimates. We therefore commit to conducting sensitivity analyses at r = 0.3 and r = 0.7 to evaluate the robustness of composite effect sizes to this assumption; results across all three values will be reported transparently in the supplementary materials.

## Primary analyses

### RO1: Model selection and justification

Given the expected heterogeneity across populations, stimulation protocols, and outcome measures, we will employ a random-effects model for all primary analyses. We will use the restricted maximum likelihood estimator (REML) as our default τ² estimator for all primary analyses. REML is preferred over the method-of-moments DerSimonian–Laird (DL) estimator because simulation studies consistently show that REML produces less biased estimates of between-study variance and yields confidence intervals with better coverage, particularly in the small-to-moderate sample sizes typical of this literature ([42];[43]). We retain DL only in the sensitivity analysis comparing estimators (see Statistical Model Sensitivity below), where its results will be presented alongside REML for transparency.

The overarching analysis will pool all eligible effect sizes comparing taVNS to control conditions across all cognitive outcomes. This analysis will yield an overall Hedges’ g with its 95% confidence interval, accompanied by z-test statistics and associated p-values. Importantly, we will report prediction intervals alongside confidence intervals when feasible, as these provide important information about the expected range of true effects in future similar studies, accounting for between-study heterogeneity.

RCTs constitute the primary evidence base for this synthesis. Non-randomised interventional studies (single-arm before–after designs) are retained as a supplementary stratum and will be analysed separately using ROBINS-I for risk-of-bias assessment and a distinct GRADE certainty rating (as described in the Quality Assessment section above); they will not be pooled with the RCT stratum in the primary analysis but will be reported alongside the primary results for contextual comparison.

### RO2: clinical population stratification

To address our second RO concerning taVNS effects in clinical populations, we will stratify studies into distinct population subgroups. These include healthy control participants, individuals with depression, patients diagnosed with ADHD, those with Alzheimer’s disease or mild cognitive impairment, participants with other neuropsychological conditions. We will conduct separate meta-analyses for each clinical population that includes at least three independent studies, ensuring sufficient power for meaningful estimation. Direct comparison between clinical and healthy populations will be performed using mixed-effects models, which allow us to test whether population type significantly moderates the effect of taVNS on cognitive functions. For clinical populations where sufficient data exist, we will calculate the number needed to treat, providing a clinically interpretable metric of intervention efficacy that complements standardised mean differences.

### Heterogeneity assessment

#### Quantifying heterogeneity.

We will employ multiple complementary statistics to characterise between-study variability. The I² statistic will quantify the percentage of total variance attributable to heterogeneity rather than sampling error, with values below 25% indicating low heterogeneity, values between 25% and 75% suggesting moderate heterogeneity, and values exceeding 75% representing high heterogeneity. Cochran’s Q test will provide a formal statistical test of heterogeneity, with a liberal significance threshold of p < 0.10 given the test’s known low power [44]. Beyond these conventional metrics, we will report tau (τ), the square root of the between-study variance, which is expressed in the same unit as the effect size metric and therefore carries direct interpretive meaning (e.g., a τ of 0.20 means that the true effects are spread with a standard deviation of 0.20 Hedges’ g units across studies; [45]). Tau-squared (τ²) will also be reported for completeness but τ will be the primary heterogeneity scale statistic discussed in the text. Finally, we will calculate 95% prediction intervals for all pooled estimates.

#### Exploring Sources of Heterogeneity.

When heterogeneity is substantial (I² > 50% or significant Q-test), we will systematically investigate potential sources through meta-regression analyses, stratified subgroup analyses, and targeted sensitivity analyses. This exploratory phase will focus on the moderator variables detailed in our RO3, examining how study characteristics, participant features, and methodological factors contribute to variation in observed effects.

### RO3: moderator analyses

To pre-empt concerns about multiplicity and interpretive ambiguity, the moderator analyses are organised into two tiers: (1) a small number of pre-specified primary moderators with strong theoretical or empirical rationale, and (2) a larger set of exploratory moderators to be interpreted with appropriate caution and as hypothesis-generating rather than confirmatory. Primary moderators are: (a) stimulation frequency (Hz) and intensity (mA), given that dose-response is the central mechanistic question; (b) clinical versus healthy population status, given that RO2 directly concerns this distinction; and (c) overall risk of bias, to assess whether methodological quality inflates effect estimates. All remaining moderators listed below are designated exploratory and will be reported with explicit acknowledgement of increased Type I error risk.

#### Continuous parameter analysis.

The dosimetric characteristics of taVNS interventions represent potentially critical moderators of treatment efficacy. We will conduct meta-regression analyses examining stimulation frequency (Hz), intensity (mA), session duration (minutes), total intervention period (days or weeks), and cumulative number of sessions as continuous predictors. For frequency and intensity, we will test both linear and quadratic relationships to capture potential non-monotonic dose-response patterns. Each continuous moderator will first be examined in univariate meta-regressions; if ten or more studies provide data for a given parameter, we will proceed to multivariate meta-regression models that can assess the independent contribution of each parameter while controlling for others. All meta-regression results will report regression coefficients with 95% confidence intervals and R² values indicating the proportion of between-study variance explained by each moderator.

#### Categorical parameter analysis.

Beyond continuous relationships, we will examine categorical distinctions in stimulation protocols through subgroup analyses. Frequency will be classified into low (≤1 Hz), medium (1–25 Hz), and high (>25 Hz) bands. Stimulation site will be categorised according to the specific anatomical target, including the cymba conchae, tragus, and other auricular locations. These categorical analyses will employ Q-tests to assess whether effect sizes differ significantly across subgroups.

#### Continuous demographic variables.

Participant characteristics may substantially influence responsiveness to taVNS. We will examine mean age (in years), percentage of female participants, and baseline cognitive performance (when reported) as continuous moderators through meta-regression.

#### Categorical demographic stratification.

Complementing continuous analyses, we will conduct subgroup analyses comparing effects across age categories (children and adolescents: ≤ 17 years; young adults: 18–35 years; middle-aged adults: 36–59 years; older adults: ≥ 60 years) and clinical severity levels (mild, moderate, and severe impairment). These categorical analyses may reveal threshold effects or qualitative differences that continuous analyses might obscure.

#### Study design Characteristics.

We will compare effects between randomised controlled trials and quasi-experimental designs, parallel group versus crossover designs, and different control conditions (active control, sham stimulation, or waitlist control).

#### Risk of bias and quality indicators.

Studies will be stratified according to their overall risk of bias rating (low risk, some concerns, or high risk) as determined by the RoB 2 assessment. This analysis will test whether study quality moderates effect size estimates, potentially indicating the presence of methodological bias. Additionally, we will examine whether effects differ according to assessment timing (immediate post-intervention, short-term follow-up of one month or less, or long-term follow-up exceeding one month), revealing the potential temporal dynamics of taVNS effects on cognition functions.

### Sensitivity analyses

#### Core robustness tests.

We will conduct a focused set of sensitivity analyses to assess the robustness of our conclusions. First, we will perform leave-one-out (LOO) analyses, systematically removing each study in turn and recalculating the pooled effect; this simultaneously identifies influential individual studies and studies whose removal substantially changes the overall estimate (i.e., acts as a data-driven outlier check), making a separate outlier-removal step redundant. Second, the trim-and-fill (TAF) method has been removed here as a general robustness test given well-documented concerns about its poor performance under heterogeneity [e.g., 46]. The primary publication-bias assessment will instead rely on PET-PEESE and selection models (see Publication Bias Analyses).

#### Statistical model sensitivity.

Beyond these substantive sensitivity analyses, we will examine the stability of our results across statistical estimators for the between-study variance. REML is our pre-specified primary estimator (see RO1 above). As a sensitivity check, we will re-run all primary analyses using the DerSimonian–Laird (DL) estimator and report the two sets of results side-by-side. If REML and DL yield substantively different conclusions, we will note this in the discussion and interpret both sets of results.

### Publication bias analyses

#### Visual inspection methods.

We will employ multiple complementary approaches to evaluate this potential bias. Visual inspection will begin with funnel plots displaying effect sizes against their standard errors, generated separately for the overall effect and each cognitive domain. We will create contour-enhanced funnel plots that overlay regions of statistical significance, facilitating identification of whether any asymmetry appears concentrated in areas of non-significance, which would strengthen the case for publication bias.

#### Statistical tests for small-study effects.

Visual inspection will be supplemented by formal statistical tests. Egger’s regression test will assess funnel plot asymmetry, with a significance threshold of p < 0.10 given the test’s known low power in small meta-analyses [46]. We have removed Begg’s rank correlation test from the protocol: the test is generally less powerful than Egger’s and the two tests address the same question redundantly; including both adds complexity without meaningful interpretive gain. Trim-and-fill has similarly been removed from the primary publication-bias assessment given simulation evidence that it performs poorly under heterogeneity and can produce systematically misleading adjusted estimates [46]. Our preferred bias-correction methods are PET-PEESE and selection models, reported in the Advanced section below.

#### Advanced publication bias methods.

When at least ten studies are available, we will conduct more sophisticated analyses. The Precision-Effect Test (PET) and Precision-Effect Estimate with Standard Error (PEESE) provide complementary approaches to adjusting effect size estimates for small-study effects, with PEESE typically preferred when PET indicates a non-zero effect [47]. We will additionally apply three-parameter selection models that formally model the publication selection process [48]. We have removed p-curve analysis from the protocol, as its diagnostic utility in the context of heterogeneous meta-analyses is questionable and its application to pooled effect sizes rather than individual study p-values can produce misleading results.

We will consider publication bias to be substantial if multiple indicators converge, specifically, if Egger’s test reaches p < 0.10, or if the adjusted effect size differs from the observed effect by more than 20%. In such cases, we will prioritise adjusted estimates in our conclusions while acknowledging the inherent uncertainty in bias correction methods.

### Additional exploratory analysis

#### Dose-response relationships.

Beyond simple linear moderator analyses, we will conduct comprehensive dose-response investigations to identify optimal stimulation parameters. These analyses will test for linear, logarithmic, and polynomial relationships between stimulation parameters and effect sizes, as biological responses often exhibit non-linear dose-response curves. If sufficient studies provide data on multiple parameters, we will explore interaction effects, such as whether the relationship between frequency and efficacy depends on stimulation intensity. These analyses may ultimately inform recommendations for optimal taVNS protocols.

#### Time-course analysis.

The temporal dynamics of taVNS effects warrant careful examination. We will compare effect sizes measured immediately after intervention to those assessed after delays, testing whether effects emerge gradually or decay over time. We will examine whether longer intervention periods produce more durable effects at follow-up, assessing whether cumulative stimulation builds lasting cognitive benefits. These time-course analyses will inform practical recommendations about intervention duration and timing of outcome assessment.

#### Baseline performance interactions.

The baseline cognitive capacity of participants may critically moderate intervention efficacy. Through meta-regression, we will test whether baseline cognitive performance predicts the magnitude of improvement following taVNS. This analysis addresses the “cognitive reserve” hypothesis, which predicts larger effects in individuals with lower baseline performance due to greater room for improvement, against the alternative possibility that higher-functioning individuals may be more responsive to cognitive enhancement interventions. Understanding these baseline interactions will guide patient selection and expectation management in clinical applications.

### Interpretation framework

#### Effect size benchmarks.

Interpreting the magnitude of cognitive enhancement effects requires contextualised judgment rather than mechanical application of generic benchmarks. Following Cohen’s conventional guidelines, we will consider effect sizes around g = 0.20 as small, g = 0.50 as medium, and g = 0.80 as large. However, we recognise that in the context of cognitive enhancement interventions, particularly for healthy populations, even small effects may have meaningful implications. Conversely, for clinical populations experiencing substantial cognitive impairment, medium to large effects may be necessary to produce clinically meaningful improvement. Our interpretation will therefore consider effect magnitude in relation to the specific population, the domain of cognition affected, and the comparison to existing interventions.

#### Minimum clinically important difference.

For clinical populations and for specific cognitive tests with established psychometric properties, we will contextualise observed effect sizes against published minimum clinically important differences (MCIDs). MCIDs are expressed in the original raw-score units of each instrument, whereas our meta-analytic effect sizes are standardised (Hedges’ g). To bridge this gap, we will apply the following procedure for each cognitive domain where an established MCID exists: (1) we will identify the most commonly used outcome measure within that domain in the included studies; (2) we will obtain the normative standard deviation for that measure from the test’s standardisation sample or from the pooled control-group SD across included studies; (3) we will convert the MCID to a standardised effect size by dividing the MCID (in raw points) by that standard deviation. The resulting standardised MCID threshold can then be directly compared to our pooled Hedges’ g. This procedure will only be applied to measures for which both a published MCID and a normative or study-level SD are available; where these are absent, we will limit interpretation to generic Cohen’s benchmarks and contextualise results relative to other cognitive interventions. All conversion steps and the SDs used will be reported transparently in the supplementary materials.

#### Quality of evidence assessment.

Beyond effect size estimation, we will rate the certainty of evidence using the GRADE (Grading of Recommendations, Assessment, Development and Evaluations) framework [38]. This structured approach considers five domains that can reduce confidence in estimates: risk of bias across studies, inconsistency (heterogeneity) of results, indirectness (extent to which the evidence addresses our research questions), imprecision (width of confidence intervals), and publication bias. Evidence begins at high certainty for bodies of randomized trials, then is rated down for serious or very serious concerns in these domains. Our final GRADE ratings will be high, moderate, low, or very low certainty, communicating not just what we found but how confident we should be in these findings.

### Statistical software and packages

All statistical analyses will be conducted using RStudio (version 2025.03.1, R version 4.4.1). The primary meta-analytic procedures will be implemented through the metafor (v4.6-0) and meta (v6.3-2) packages for random- and mixed-effects modelling, effect size computation, and heterogeneity assessment. For robust variance estimation (RVE) and cluster-robust sensitivity analyses, we will employ the *clubSandwich* (v0.6.0) and *robumeta* (v3.1) packages. Moderator and meta-regression analyses will be performed using the *metafor* framework with REML estimation. For generating contour-enhanced funnel plots and forest plots, we will use *ggplot2* (v3.5.1) and *ggpubr* (v0.6.0).

## Discussion

The present systematic review and meta-analysis protocol aims to synthesise and critically evaluate the current evidence on the effects of taVNS on cognitive functions. Existing studies vary considerably in population characteristics, stimulation parameters, and methodological quality, producing a fragmented evidence base that limits clinical translation. By integrating data across healthy and clinical populations, we aim to delineate not only the general efficacy of taVNS but also the domain-specific and population-dependent patterns of its cognitive influence.

### Potential theoretical and mechanistic implications

Within the conceptual “Sorting Hat” framework proposed in this protocol; cognitive functions are understood as an integrated system composed of interdependent functional domains. taVNS, by virtue of its widespread neural projections, may exert differentiated yet convergent influences across these domains, strengthening working memory and attention, enhancing emotional regulation and social cognition), promoting adaptive flexibility and learning, and optimising executive functioning and decision-making. This integrative approach transcends the reductionist tendency of prior research, recognising that cognitive processes operate as an orchestrated network rather than isolated faculties.

This protocol adopts several potential methodological advancements to enhance the validity and interpretability of results. First, by including both healthy and clinical populations, it aims to bridge the gap between mechanistic and translational research and enable the examination of whether taVNS exerts baseline-dependent effects—enhancing cognition in impaired populations while fine-tuning neural efficiency in healthy individuals.

Second, the comprehensive moderator analyses proposed, including stimulation parameters (frequency, intensity, duration), participant demographics, and study design variables, will provide critical insights into the boundary conditions of taVNS efficacy. Such analyses can inform the standardisation of stimulation protocols, a current limitation in the field.

Furthermore, the inclusion of dose–response and time-course analyses will represent a significant step toward identifying optimal stimulation “dosage” and understanding the temporal dynamics of taVNS effects. The use of the GRADE framework to assess the overall certainty of evidence will also provide clinicians and researchers with a clear interpretative benchmark. If the forthcoming meta-analytic synthesis identifies consistent and clinically meaningful improvements, taVNS could be positioned as a cost-effective, safe, and accessible adjunct for cognitive rehabilitation.

At the same time, exploring its effects in healthy and subclinical populations can shed light on its potential as a cognitive enhancer—a topic with growing ethical and societal relevance. Establishing empirical boundaries between therapeutic benefit and enhancement will inform future policy, clinical guidelines, and research ethics.

## Conclusion

This protocol outlines a comprehensive, theoretically grounded, and methodologically rigorous plan to synthesise the effects of taVNS across diverse populations and cognitive domains. By integrating mechanistic insights with systematic evidence synthesis, it seeks to move the field beyond fragmented findings toward a cohesive understanding of how vagal stimulation can shape human cognition.

### Patient and public involvement statement

Patients or members of the public were not involved in the design, conduct, reporting, or dissemination plans of this research. Because this study is a systematic review and meta-analysis protocol based solely on data extracted from previously published studies, no direct patient recruitment or public participation was required. Nevertheless, the review is designed with clear translational intention to inform future clinical applications of taVNS for cognitive enhancement and rehabilitation in both healthy and clinical populations. To ensure that future stages of this research remain patient-centred, the findings from the completed review will be disseminated in accessible formats through public-facing summaries and infographics and shared with patient advocacy and neuromodulation interest groups. This approach aims to bridge the gap between mechanistic research and real-world clinical understanding, ultimately contributing to evidence-based and ethically grounded use of taVNS in cognitive health interventions.

## Supporting information

S1 Appendix Full electronic search strategies. Complete search strings for all six electronic databases (PubMed, EMBASE, PsycINFO, Web of Science Core Collection, CENTRAL, and Scopus), including all Boolean operators, controlled vocabulary terms (e.g., MeSH headings), and field tags used to identify studies of transcutaneous auricular vagus nerve stimulation and cognitive outcomes [49–53].
(DOCX)

S2 Appendix PRISMA-P checklist. Completed Preferred Reporting Items for Systematic Review and Meta-Analysis Protocols (PRISMA-P) 2015 checklist indicating where each item is addressed in the manuscript.
(DOCX)
