# Supplementary material for: Sorting the mind: A systematic review and meta-analysis protocol of transcutaneous auricular vagus nerve stimulation on cognitive functions
Source: PLoS One. 2026 Apr 24;21(4):e0347849. doi: 10.1371/journal.pone.0347849 (PMC13108857; doi:10.1371/journal.pone.0347849)
Supplement: S1 Appendix — Complete search strings for all six electronic databases (PubMed, EMBASE, PsycINFO, Web of Science Core Collection, CENTRAL, and Scopus), including all Boolean operators, controlled vocabulary terms (e.g., MeSH headings), and field tags used to identify studies of transcutaneous auricular vagus nerve stimulation and cognitive outcomes [49–53]. (DOCX) [file pone.0347849.s001.docx]

**Appendix I- Table 1- Search terms**

| taVNS | Executive Functions | Working Memory and Attention | Social Cognition and Emotional Regulation | Cognitive Flexibility and Learning | Cognitive Impairment and Disorders |
| --- | --- | --- | --- | --- | --- |
| transcutaneous auricular vagus nerve stimulation | executive function* | working memory | social cognition | cognitive flexibility | cognitive impairment |
| taVNS* | decision-making | short-term memory | emotion regulation | learning ability | Alzheimer’s disease |
| auricular vagus nerve stimulation | impulse control | attention | emotion recognition | adaptability | ADHD |
| transcutaneous vagus nerve stimulation | planning | sustained attention | social interaction | task-switching | Attention Deficit Hyperactivity Disorder |
| external vagus nerve stimulation | problem-solving | focused attention | emotional intelligence | set-shifting | depression |
| vagus nerve stimulation auricula | self-regulation | selective attention | affective regulation | information processing | neurodegenerative disorder* |
|  | inhibition | divided attention | emotional control | learning strategies | dementia |
|  | cognitive flexibility | n-back task | social decision-making | problem-solving adaptability | memory disorders |
|  | cognitive control | Stroop tes |  |  | mild cognitive impairment |
|  |  | digit span |  |  | executive dysfunction |
|  |  | Go/no-go |  |  | neuropsychological disorders |
|  |  |  |  |  |  |
