# Supplementary material for: Sorting the mind: A systematic review and meta-analysis protocol of transcutaneous auricular vagus nerve stimulation on cognitive functions
Source: PLoS One. 2026 Apr 24;21(4):e0347849. doi: 10.1371/journal.pone.0347849 (PMC13108857; doi:10.1371/journal.pone.0347849)
Supplement: S2 Appendix — Completed Preferred Reporting Items for Systematic Review and Meta-Analysis Protocols (PRISMA-P) 2015 checklist indicating where each item is addressed in the manuscript. (DOCX) [file pone.0347849.s002.docx]

# **PRISMA-P 2015 Checklist**

*Preferred Reporting Items for Systematic Review and Meta-Analysis Protocols*

Manuscript: Sorting the Mind: A Systematic Review and Meta-Analysis Protocol of Transcutaneous Auricular Vagus Nerve Stimulation on Cognitive Functions

| **Section/Topic** | **Item #** | **Checklist Item** | **Location** |
| --- | --- | --- | --- |
| **ADMINISTRATIVE INFORMATION** |  |  |  |
| **Title** |  |  |  |
| Identification | 1a | Identify the report as a protocol of a systematic review | Title (p.1): "...Systematic Review and Meta-Analysis Protocol..." |
| Update | 1b | If the protocol is for an update of a previous systematic review, identify as such | N/A - This is not an update |
| Registration | 2 | If registered, provide the name of the registry and registration number | Protocol and Registration (p.6): PROSPERO ID: CRD420251166774 |
| **Authors** |  |  |  |
| Contact | 3a | Provide name, institutional affiliation, and e-mail address of all protocol authors; provide physical mailing address of corresponding author | Author Information (p.1): Fangqing Liu, Faculty of Biology, Medicine and Health, The University of Manchester; fangqing.liu@postgrad.manchester.ac.uk |
| Contributions | 3b | Describe contributions of protocol authors and identify the guarantor of the review | To be added in final submission |
| Amendments | 4 | If the protocol represents an amendment of a previously completed or published protocol, identify as such and list changes; otherwise, state plan for documenting important protocol amendments | N/A - No amendments; amendments will be tracked via PROSPERO |
| **Support** |  |  |  |
| Sources | 5a | Indicate sources of financial or other support for the review | Funding Statement (p.1): No funding received |
| Sponsor | 5b | Provide name for the review funder and/or sponsor | N/A - No sponsor |
| Role of sponsor/funder | 5c | Describe roles of funder(s), sponsor(s), and/or institution(s), if any, in developing the protocol | N/A - No external funding |
| **INTRODUCTION** |  |  |  |
| Rationale | 6 | Describe the rationale for the review in the context of what is already known | Introduction (pp.1-6): Current Evidence Landscape; The Present Study |
| Objectives | 7 | Provide an explicit statement of the question(s) the review will address with reference to participants, interventions, comparators, and outcomes (PICO) | Research Objectives (p.6): RO1, RO2, RO3; Table 1: PICOS criteria |
| **METHODS** |  |  |  |
| Eligibility criteria | 8 | Specify the study characteristics (e.g., PICO, study design, setting, time frame) and report characteristics (e.g., years considered, language, publication status) to be used as criteria for eligibility for the review | Table 1 (pp.7-8): Inclusion and Exclusion Criteria for Study Selection |
| Information sources | 9 | Describe all intended information sources (e.g., electronic databases, contact with study authors, trial registers, or other grey literature sources) with planned dates of coverage | Search Strategy (pp.6-7): Seven databases, trial registries, grey literature; inception to October 2025 |
| Search strategy | 10 | Present draft of search strategy to be used for at least one electronic database, including planned limits, such that it could be repeated | Search Strategy (p.7); Appendix I |
| **Study records** |  |  |  |
| Data management | 11a | Describe the mechanism(s) that will be used to manage records and data throughout the review | Study Selection and Data Extraction (p.7) |
| Selection process | 11b | State the process that will be used for selecting studies (e.g., two independent reviewers) through each phase of the review (i.e., screening, eligibility, and inclusion in meta-analysis) | Study Selection and Data Extraction (p.7): Two reviewers independently screening |
| Data collection process | 11c | Describe planned method of extracting data from reports (e.g., piloting forms, done independently, in duplicate), any processes for obtaining and confirming data from investigators | Study Selection and Data Extraction; Table 2 (pp.7-9) |
| Data items | 12 | List and define all variables for which data will be sought (e.g., PICO items, funding sources), any pre-planned data assumptions and simplifications | Table 2 (pp.8-9): Study Characteristics and Data Extraction Framework |
| Outcomes and prioritization | 13 | List and define all outcomes for which data will be sought, including prioritization of main and additional outcomes, with rationale | Table 2; Primary Analyses (pp.8-11): Four cognitive domains |
| Risk of bias in individual studies | 14 | Describe anticipated methods for assessing risk of bias of individual studies, including whether this will be done at the outcome or study level, or both; state how this information will be used in data synthesis | Quality Assessment (p.9): RoB 2 tool; assessments at result level |
| **Data synthesis** |  |  |  |
|  | 15a | Describe criteria under which study data will be quantitatively synthesized | RO1: Model Selection and Justification (pp.10-11) |
|  | 15b | If data are appropriate for quantitative synthesis, describe planned summary measures, methods of handling data, and methods of combining data from studies, including any planned exploration of consistency (e.g., I², Kendall's tau) | Effect Size Imputation and Calculation (pp.9-10); Heterogeneity Assessment (pp.11-12): Hedges' g, random-effects model, I², τ², Q-test |
|  | 15c | Describe any proposed additional analyses (e.g., sensitivity or subgroup analyses, meta-regression) | RO3: Moderator Analyses (pp.11-13); Sensitivity Analyses (p.13) |
|  | 15d | If quantitative synthesis is not appropriate, describe the type of summary planned | N/A - Quantitative synthesis planned |
| Meta-bias(es) | 16 | Specify any planned assessment of meta-bias(es) (e.g., publication bias across studies, selective reporting within studies) | Publication Bias Analyses (pp.13-14): Funnel plots, Egger's test, Begg's test, trim-and-fill |
| Confidence in cumulative evidence | 17 | Describe how the strength of the body of evidence will be assessed (e.g., GRADE) | Quality of Evidence Assessment; GRADE (p.14) |

*Reference: Moher D, Shamseer L, Clarke M, et al. Preferred reporting items for systematic review and meta-analysis protocols (PRISMA-P) 2015 statement. Systematic Reviews. 2015;4:1. doi:10.1186/2046-4053-4-1*
